# Pure Red Cell Aplasia with Adult Onset Still's Disease

**DOI:** 10.1155/2013/308342

**Published:** 2013-09-02

**Authors:** Nicholas Robillard, Paul Van Nguyen, Robert Wistaff, Mikhael Laskine

**Affiliations:** Internal Medicine Service, Department of Medicine, Hotel-Dieu Hospital, University Hospital of Montreal (CHUM), 3840 St. Urbain Street, Montreal, QC, Canada H2W 1T8

## Abstract

Adult Onset Still's Disease (AOSD) is a rare inflammatory syndrome mostly seen in young adults. Known for its wide range of clinical manifestations, AOSD often presents with nonremitting systemic signs and symptoms. Many rare case associations have been described with AOSD, but only few with pure red cell aplasia (PRCA). We are presenting a fourth known case of a young female adult with AOSD and PRCA in the literature.

## 1. Introduction

 Pure red cell aplasia (PRCA) is a very rare disease characterized by a severe anemia without a normal reticulocyte response and with the near complete absence of erythroid precursors in the bone marrow [1]. Although this disease is rather uncommon, many etiologic associations with it have been established. Drugs, infections [2, 3], immune disorders, lymphoid malignancies, myeloid malignancies, and certain forms of cancer [4] are just some of the recognized causes of PRCA. Even connective tissue diseases (rheumatoid arthritis [5, 6], systemic lupus erythematosus [7, 8], etc.) have been incriminated as causal agents.

 An uncommon rheumatic disease is Adult Onset Still's Disease (AOSD). Its incidence is believed to be of 16 cases per 10 000 000 persons in a retrospective study by a French group in 1995 [9]. AOSD is known for its wide range of presentations, making the rapid diagnosis of this disease difficult. To palliate this clinical problem, many sets of criterion have been proposed by different groups. A study by Masson et al. [10] in 1996 came to the conclusion that the Yamaguchi criteria [11] had the best sensitivity to establish the diagnosis of AOSD.

 Hematologic complications of this syndrome are diverse: reactive hemophagocytic syndrome [12], diffuse intravascular coagulation, microangiopatic hemolytic anemia, and thrombotic thrombocytopenic purpura/hemolytic uremic syndrome [13]. An uncommon association with AOSD is that of a PRCA [14–16].

 The following case report will present the story of a patient with pure red cell aplasia related to an Adult Onset Still's Disease.

## 2. Case Report

 A 34 year-old Vietnamese woman presented to the Hôtel-Dieu Hospital in Montreal in autumn of 2006. Her main complaints were loss of energy, fatigue, loss of weight and fever. This patient had immigrated to Canada 4 months ago. She had no prior known illnesses. She was without allergies and was not taking any prescription drugs. 

 Further history of the patients revealed the presence of a fever of over 38,5°C for the past month and a half. She had started to feel tired at the same time. She also noted arthralgia to her wrist, ankles, and knees. The patient noted no other rheumatologic symptoms. The remaining history was uneventful. She had not been exposed to infectious agents in Vietnam and did not recall seeing any of the same symptoms in her next of kin. 

 On physical examination, the patient had pale colored skin and had slight dyspnea. She was not toxic. Her vital signs were normal apart from a 112 beat per min tachycardia. Her conjunctivas were pale, but the rest of her neck and head exam was normal. Her cardiopulmonary evaluation was also normal. The evaluation of her abdomen revealed a hepatosplenomegaly (confirmed on her abdominal ultrasound and scans). The only other relevant finding was a synovitis of her wrists, ankles, knees, and metacarpal articulations of both hands. No rash was documented.

To our surprise, her initial blood tests showed a severe anemia with 19 g/L of hemoglobin, which was confirmed with a second analysis. They also demonstrated a leucocytosis (total white blood cells count of 23.0 × 10^9^/L), and slightly elevated mean corpuscular erythrocyte volume of 106 fl. Platelets were of normal range. Other abnormalities included perturbed levels of AST of 1442 U/L (normal: 13–39) and ALT of 737 U/L (normal: 8–31). She had an inflammatory syndrome with C-reactive protein at 77.40 mg/L, sedimentation rate at 102 mm/H, and the protein electrophoresis marked by severe inflammation. Finally her ferritin was extremely elevated: 25,673 *μ*g/L (normal: 11–90 *μ*g/L). Septic workup was negative and no bacterial growth was apparent in all of the hemocultures. As PRCA is being suspected, finding the etiology became a priority for the medical team. However, after extensive testing, none of the common causes of PRCA were found: no infections (negative serology for parvovirus B19, EBV, CMV, viral hepatitis, HIV, etc.) and no evidence of neoplasms were found on cerebral, thoracic, and abdominal CAT scans and an autoimmune workup only found a slightly elevated ANA of 1/320 that normalized in the following analyses.

**Figure 1:**
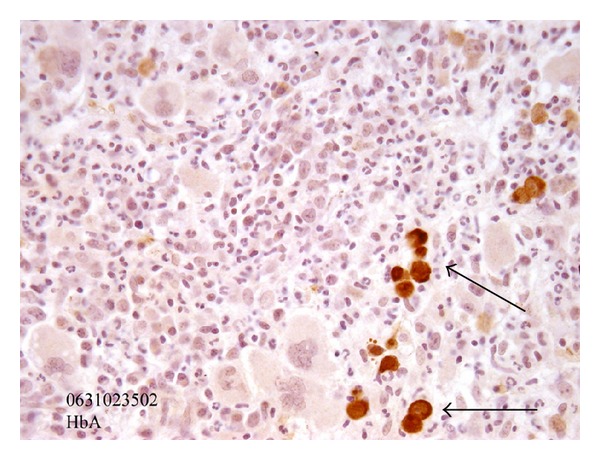
Bonne marrow biopsy before treatment (immunohistochemistry by antibody directed against HbA). Almost complete absence of the red cell lineage (see arrows).

 A bone marrow biopsy was then undertaken (Figure 1). It was compatible with the presence of a PRCA: almost complete absence of the red cell lineage. It also displayed a hypercellularity, hyperplasia of the granulocytic precursors and increased histiocytes on CD68 staining. The reticulocyte response was suboptimal at levels of 8 × 10^9^/L (normal 0 to 110 × 10^9^/L).

 Parallel to the diagnostic of PRCA, an Adult Onset Still's disease was suspected. The presence of non-remitting systemic symptoms (fever and weight loss) and the presence of 3 major criteria (fever, arthralgia/arthritis and persistent leucocytosis) and 4 of the minor criteria (lymphadenopathy, hepatosplenomegaly, elevated serum hepatic aminotransferases, and the absence of multiple occasions of an ANA, anti-DNA or RF) from the Yamaguchi criterions combined with the absence of any other rheumatic diseases or neoplastic diseases lead us to conclude on the presence of an AOSD. Of note, the patient often presented levels of ferritin >100,000 *μ*g/L during her relapses. 

**Figure 2:**
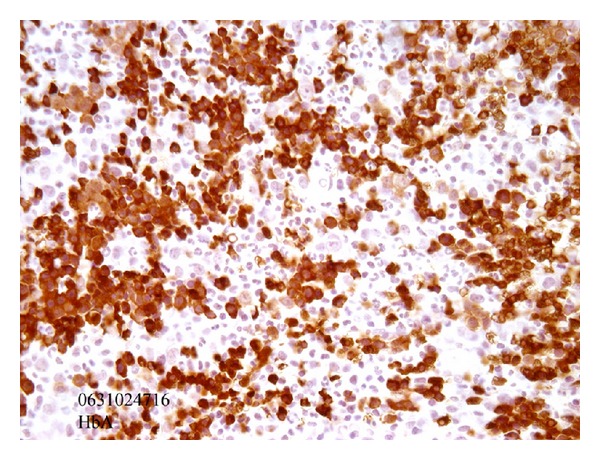
Bonne marrow biopsy after 2-week treatment with cortisone (the same immunohistochemistry analysis as in Figure 1). Complete normalization of the red cell lineage.

 The patient was then treated with high doses of corticosteroids. This led to the complete normalization of her bone marrow on a second biopsy performed after a two-week course of oral cortisone (Figure 2). She was then placed on a weaning protocol and sent home.

## 3. Discussion

 Presented here was a case of Still's disease associated with Pure Red Cell Aplasia. As mentioned earlier, the diagnosis of the AOSD was based on the absence of other more likely diagnostics and the presence of at least five Yamaguchi criteria [11]. The patient also demonstrated extremely high levels of ferritin, a characteristic often encountered with Still's disease. Furthermore, the patient's bone marrow evaluation was compatible with some of the most frequent findings of Min et al. [17] in 2003 after the evaluation of bone marrow biopsies of 12 consecutive patients with AOSD. These findings were granulocyte hyperplasia (100%), hypercellularity (75%), and histiocytosis (25%). Other possible findings that our patient did not present are reactive hemophagocytosis (16.7%) and plasmacytosis (8.3%).

Initial levels of ANA were slightly elevated at a level of 1/300 in this patient. A hallmark of Still's disease is the absence of autoimmune antibodies. However, it has been reported that up to 10% of patients with AOSD can have a weak positive titer for ANA or RF [18]. The low initial levels of ANA in this patient do not contradict the diagnosis of AOSD to our view because of the normalization of this titer even before therapeutic initiation. In addition, the patient's presentation does not correlate with any other rheumatologic disease.

The diagnosis of a PRCA was confirmed here by the combination of a profound anemia, absence of reticulocyte response, and presence of severely depleted stock of red blood cell progenitors on marrow examination. All possible aetiologies were excluded after intensive testing. The sole diagnostic association left possible was that of a PRCA caused by an Adult Onset Still's Disease. Emphasizing this causal relationship is the patient's quick response to high doses of corticosteroids. Within two weeks, her bone marrow was back to normal.

 Thus to conclude, it is in our opinion that this patient presented a case of Pure Red Cell Aplasia in the setting of an Adult Onset Still's Disease. Very few cases of rheumatic disease associated PRCA have been published, even less when AOSD is implicated. To our knowledge, this is the fourth described case in the medical literature [14–16].
